# Burdett's Hospitals and Charities, 1907

**Published:** 1907-03-30

**Authors:** 


					470 THE HOSPITAL. March 30, 1907.
""'RDETT'S HOSPITALS AND CHARITIES, 1907.
We are requested to point out that an error has occurred in the table on page 165 in regard to the figures of Guy's
Hospital, which will affect the totals in the table on page 172. The figures on page 165 should read as follows :?
Hospitals with Medical Schools.?Income, 1905.
Name.
be?
Wo.
3
a os
tvo
Km
? >>
cfi 03
? ta
? r-
III
c 2
?ph
c -e v
oca
OS o
fasLi. .
IS? 118
" ss
c S
? 5
>!
oj ^
Oh Ph
-cE
te V w
HO?
Extraordinary.
5.3
3 '3 <u
Mkdical Schools.
London. ? I ? I ?
Account
1! London
? j St. Bartholomew
31 Guy's
4?) St. Thomas's ....
5 i St. George's
61 Middlesex j Cancer Win.,a ...
7 j St. Mary's
8! University College IIos >ital
9 King's College Hospital
10! Westminster Hospital
11 j Charing Cross j 2,43S
12, Koj-al Free  ;.... ! 1,400
14,549, 13,90b! 82
? I 412! ?
3,377! 3,026; ?
919 879; 1
6.160 2,149i 82
2,368' 1,503 438
'96 1,212: ?
4,889' 3,858: 162
2,316 3,487! 31
2,032   ~
1,660
3,309
2,339 91
? ?
8,000; 6,769
5T000! 2,399
2,000' 2^292
1,000 3,236
? | 333
2,000; 2,981
4,903 366
9,053, 40
2,000
2,000
2,750
1,500
1,500
2,084
1,811
1,900 345
1,401 295
?
1,166
340
180
333
500
100
542
365
395
1,484; 286
43,352 50,050 1,781 27,750 , 26,690' 4,897
? ?
1,798 15,493
66,423
36,534
46,592
13,614
5,301
1,905
2,788
3,633
4,354
3,441
2,392
1,223
?
5,899
500
1^284
61
775 1,956
1,089'
1,493
5
50
100
36
4,698
276
235
49
6
166
154
70
144
36
47
183
13
199
1,793; 203,723 7,744 3,548^ 7,220^ 1,012 379,565
?
70,392
68,590
57,367
50,285
26,780
14.495
4,246
17.496
14,062
16,200
19,212
11,912
8,528
?
2,500
31,480
54,082
1,250
1,050
14,145
1,467
4,000
2,000
6,317
1,654
? I
22,900 : 1
92 2
10,851
873,690
10,016
15,795 \
1,193 r
10,359
5,452
47,046
3,750
7,268
5,885
119,945 214,297
The totals on page 172 should read as follows :?
Sources, ancl Amounts of Income of Metropolitan, Provincial, Scotch, and Irish General Hospitals, with percentages
.which each Sub-head is of the Total Ordinary Income for the year 1905.
ws
W?
OO
ondon...
s s
|s
Qa
c 5
5 ?
? >>
?
63,40813.3 86,846 18.3 38,050
rovincialj 148.060 26!9! 8^3931^5 ?
38,527 23.4 11,999 8.9 ?
Jotch
ish
4,338 16.4
254,863
8C6 2.7 ?
I I
?
8.0 36,003 7.6 7,025 1.5
? a "2
S 2i
?
1,938
6 Ot
e 2
2 as
Sps
s ts g
Ph
10.9
? i 44,268 8.0 42,857 7.7 60,564
? 3,909 2 9 ? i ? 28,636 21.1
? 727 2.5 922 3.1 3,577 12.0
? ?
0.4:216,767,45.6 7.922
142,775
39,857
b14,936
25.8
29.3
50.4
I II I
21.3 ;i85,C44 15.5 38,050 3.2 84,907 7.1'50,?04 4.3 94,715 8.0 414,335 34.7
I I ' I : I I I |
8,700
26
177
0.6
$ 05 ?
VS to
3 ? %
?5Ph Pm
?
4,095
3,660
598
427
16,825! 1.4 8,780
Sc -g
C g ' S
a ?
'-5 >>
I ?
0.9 12,070
0.7| 9,826
0.4 5,273
1.4; 2,591
0.7; 29,760
is
~ A
?
1,484! 0.3
6,223. 1.1
3.9 6,918 5.1
8.7
2.5
638 2.2
15,263, 1.3
*E?-
2 a
II
Extraordinary
Income
? P U I to
>2 ?
? rs - cj
?
475,608
552,326
135,743
29,669
1,193,346
?
144,144
169,701
34,702
2,223
?
237,510
307,714
58,773
2,968
350,770 606,965
p 6 would suggest that subscribers to Burdett's " Hospitals
and Charities," 1907, should cut the above tables out and
insert them in their copy of the book, so that they may
have the correct figures in the most handy form for per-
manent reference.

				

